# High-protein diet scores, macronutrient substitution, and breast cancer risk: insights from substitution analysis

**DOI:** 10.1186/s12905-024-02959-7

**Published:** 2024-02-16

**Authors:** Mitra Kazemi Jahromi, Hamid Ahmadirad, Hossein Farhadnejad, Mostafa Norouzzadeh, Ebrahim Mokhtari, Farshad Teymoori, Niloufar Saber, Zeinab Heidari, Parvin Mirmiran, Bahram Rashidkhani

**Affiliations:** 1 Endocrinology and Metabolism Research Center, Hormozgan University of Medical Sciences, Bandar Abbas, Iran; 2 Nutrition and Endocrine Research Center, Research Institute for Endocrine Sciences, Shahid Beheshti University of Medical Sciences, Tehran, Iran; 3 Department of Community Nutrition, School of Nutrition and Food Sciences, Shiraz University of Medical Sciences, Shiraz, Iran; 4 Department of Nutrition, School of Public Health, Iran University of Medical Sciences, Tehran, Iran; 5 Student Research Committee, Ahvaz Jundishapur University of Medical Sciences, Ahvaz, Iran; 6 Department of Community Nutrition, Faculty of Nutrition Sciences and Food Technology, National Nutrition and Food Technology Research Institute, Shahid Beheshti University of Medical Sciences, Tehran, Iran

**Keywords:** Dietary patterns, High-protein, Breast cancer, Adult

## Abstract

**Background:**

Evidence from recent studies suggested that variation in the quantity and quality of macronutrients in the diet may potentially play a role in predicting the risk of breast cancer (BC). In the current study, we aimed to assess the association of different high-protein diet scores and replacing fats and carbohydrate (CHO) with protein in the diet with the BC risk among Iranian women.

**Methods:**

The current hospital-based case-control study was conducted on 401 participants, aged ≥ 30 years old, including 134 women in the case group who had been diagnosed with histologically confirmed BC and 267 women in the control group. Dietary intake data was collected using a validated food frequency questionnaire, and high protein diet scores were determined. Logistic regression models were used to determine the odds ratios (OR) and 95% confidence interval (CI) of BC across tertiles of high protein diet scores. Also, we assessed how substituting protein with other macronutrients affected BC odds while adjusting for the various confounding variables.

**Results:**

Participants’ mean ± SD of age and body mass index were 47.9 ± 10.3 years and 29.4 ± 5.5 kg/m^2^, respectively. The scores of high-protein-low-CHO and fat diet, high-protein and CHO-low-fat diet, and high-protein and fat-low-CHO diet in participants were 16.5 ± 3.8, 16.5 ± 6.7, and 16.4 ± 5.9, respectively. In the multivariable model, individuals in the highest tertile of high-protein-low-CHO and fat diet score (OR:0.71;95%CI:0.56–0.90) and high-protein and CHO-low-fat diet (OR:0.76;95%CI:0.60–0.97) had lower odds of BC compared to those in the lowest tertile (*P* < 0.05). However, no significant association was found between high-protein and fat-low-CHO diet and BC risk. Our results showed that replacing fat by protein (OR_differences_:-0.40;95%CI:-0.73,-0.07) and also replacing refined-CHO by plant protein (OR_differences_:-0.66;95%CI:-1.26,-0.07) in the diet are associated inversely with risk of BC(*P* < 0.05).

**Conclusions:**

The results of our study suggested that higher adherence to a high-protein-low-CHO and fat diet, characterized by a higher intake of plant proteins and a lower intake of refined grains and saturated fat can play a protective role against the odds of BC.

## Background

Breast cancer (BC) is a common malignancy affecting both sexes, with a higher incidence in women, causing a quarter of women’s cancers and affecting 1.5 million women annually [1]. In Iran, BC ranks as the third leading cause of death among women, with a ten-year survival rate of 58.1% [2, 3]. BC impacts 8,000 Iranian women annually, and one-third of cases occur in women under 30 years old [4]. While early diagnosis of BC can improve the treatment process, its metastatic and multifactorial nature makes it difficult to treat effectively [5]. Consequently, healthcare systems bear a greater economic burden [6] and BC patients face premature death and reduced quality of life [7].

Genetic predisposition, sex, aging, unhealthy lifestyle, and poor diet are risk factors for predicting BC risk [8, 9]. Crucial preventable causes of BC mortality include dysglycemia, obesity, alcohol, and red meat consumption. These factors are directly or indirectly linked to dietary choices [10]. Therefore, in the past decades, several studies focused on the role of diet in predicting the risk of BC at different levels, including food patterns, food groups, and nutrients [11, 12].

Dietary protein intake can impact cancer risks depending on the type and amount of protein consumed [13–15]. The higher red meat consumption, as a source of dietary protein, is responsible for 3.21% of BC mortality [10]. It has been suggested that a high intake of protein could increase the risk of certain types of cancer, including prostate cancer, esophageal cell carcinoma, and colon cancer [13–15]. There are conflicting results from various studies. High-protein diets have been linked to an increased risk of respiratory tract and renal cell cancer [16, 17], but a decreased risk of prostate cancer [18]. However, there is no clear link between high protein diet and the incidence or mortality of BC [19]. Studies have suggested that a higher intake of protein can improve the survival rate in individuals with BC [20–23]. However, a long observational study has highlighted that the source of protein consumed is a more crucial factor in determining the incidence of BC than the overall amount of protein consumed [24]. Notably, increased consumption of animal-based proteins may lead to a heightened risk of BC up to 20% [25].

Considering conflicting results in current research, the rising prevalence of BC in Iran, and the absence of conclusive findings on the association between the quantity and quality of dietary protein and BC risk in the Middle East and North Africa region, our objective was to explore the potential relationship between high protein diet scores and odds BC in Iranian adults. Also, we employed substitution models and compared different dietary protein sources with odds of BC.

## Materials and methods

### Study population

We conducted a hospital-based case-control study using the Shohada and Imam Hossain hospitals as referral centers in Tehran, Iran from September 2015 to February 2016. The case group consisted of 136 newly diagnosed women with BC who were aged ≥ 30 years, had been diagnosed with BC within the previous 6 months, and were not undergoing any cancer treatments at the time of the interview. For the case group, we applied a set of exclusion criteria, including individuals who followed specific dietary habits, those with a history of hormone replacement therapy (HRT), and pregnant or lactating women. The control group included 272 women, aged ≥ 30 years, who were admitted to the same hospitals during the study period for non-neoplastic conditions, such as traumas and orthopedic disorders, disk disorders, acute surgical conditions, and eye, nose, ear, or skin disorders. Also, for the control group, we excluded individuals with a history of HRT or benign breast disease, physician-diagnosed cancer in any site, and those who were pregnant or lactating, as well as those who followed special dietary habits due to a particular disease or for weight loss purposes.

Of the subjects approached for participation in the study, less than 8% declined to be interviewed. Seven participants were excluded from the analysis due to reported energy intakes that deviated by more than ± 3 standard deviation (SD) from the mean energy intakes of the population, which included five subjects in the control group and two subjects in the case group. The exclusion of participants with extreme deviations in reported energy intakes was based on the assumption that these values were not representative of their actual intake and could introduce significant variability in the analysis. By excluding these outliers, we aimed to ensure the inclusion of participants with plausible energy intakes and minimize the potential bias caused by extreme values. Finally, a total of 401 participants (134 cases and 267 controls) remained for the final analysis.

### Dietary assessment

To assess dietary intake during the year before diagnosis for cases or interviews for controls, a validated and reliable semi-quantitative 168-item food frequency questionnaire (FFQ) with standard serving sizes was used [26]. Participants were asked by skilled dietitians to report how frequently they consumed each food item on a daily, weekly, monthly, or yearly basis over the course of the previous year. The portion sizes of consumed foods were then converted into daily grams using household measures [27]. The energy and nutrient intake were computed using the United States Department of Agriculture (USDA) food composition table (FCT). The Iranian FCT was used for local food items not listed in the USDA FCT [28]. Adjustments were made to ensure compatibility and consistency between the two databases. By incorporating the Iranian Food Composition Table, we aimed to accurately estimate the nutrient composition of traditional Iranian foods in our analysis. It should be noted that due to religious considerations and legal restrictions in Iranian society, we could not collect data on alcohol consumption in participants, and therefore, it was not included in the analysis.

We calculated the main protein, carbohydrate (CHO), and fat subgroups based on their dietary sources as follows: Refined CHOs were defined as the total CHOs consumed from refined grains, sweets, and sweet snacks and drinks with added sugar. Carbohydrates obtained from other sources such as whole grains, dairy, fruits, and vegetables were considered to be non-refined CHOs. Moreover, protein and fat were classified into two categories: animal and plant sources. Animal protein was defined as dietary protein obtained from animal sources such as meat, poultry, fish, eggs, and dairy products, while plant protein was defined as protein obtained from plant sources such as legumes, nuts, seeds, and whole grains. Animal fat was determined as dietary fat obtained from animal sources such as meat, butter, cheese, and other dairy products, while plant fat was defined as fat obtained from plant sources such as nuts, seeds, and vegetable oils. In the current study, the breakdown of dietary subgroups, including refined CHOs, non-refined CHOs, animal protein, plant protein, and fat sources was made based on established nutritional classifications and their potential relevance to chronic diseases such as breast cancer risk [29–31]. These categorizations allow for a more detailed analysis of the impact of different types of CHOs, proteins, and fats on breast cancer outcomes.

### High protein diet scores definition

To determine the dietary intake of protein, fat, and CHOs, the percentage of energy intake from each nutrient was calculated and categorized into deciles. A score of 1 to 10 was assigned to each decile for the high protein, high fat, and high CHO diets, respectively. Conversely, for the low-fat and low-CHO diets, a score of 10 to 1 was assigned to each decile, respectively. The scores for each nutrient were then summed to create three types of high-protein diets, including (1) high protein- low CHO and low-fat diet, (2) high protein- high CHO and low-fat diet, and (3) high protein- low CHO and high-fat diet.

### Assessment of non-dietary exposures

Body weight was assessed using a digital scale (Seca, Germany) with a precision of 0.5 kg. Participants were instructed to wear light clothing and no shoes during the measurement. Height was measured to the nearest 0.5 cm using a tape meter that was fixed to a wall. This protocol was followed across all data collection sessions, and trained personnel conducted the measurements to minimize potential variations introduced by different individuals. The body mass index (BMI) was calculated as weight (kg) divided by height in square meters (m^2^).

Trained dietitians conducted all other questionnaires and measurements. General questionnaires were used to collect participants’ socio-demographic, lifestyle, and clinical information, including age (years), age at menarche (years), age at first pregnancy (years), smoking status (yes, no), marital status (single, married, divorced, widowed), menopausal status (pre-menopause, post-menopause), education level (illiterate, less than a high school diploma, high school diploma and more), abortion history (yes, no), number of live births (number), breastfeeding history (month), history of HRT (yes, no), history of oral contraceptive pill (OCP) use, history of benign breast diseases (yes, no), family history of cancer (yes, no), family history of breast cancer (yes, no), bra-wearing habits (day (yes, no), night (yes, no)), vitamin D supplementation (yes, no), and use of anti-inflammatory medications (yes, no). The physical activity levels of participants were also assessed using a reliable and validated questionnaire [32], and the results were reported as Metabolic Equivalents hours per week (MET-h/week) [33, 34].

To determine the socio-economic status (SES) score [35] of participants, three variables were used: family size (classified as ≤ 4 or > 4 people), education (categorized as academic or non-academic), and occupation (classified as employed or not employed). For each participant, a score of either 1 (if their family had ≤ 4 members, had an academic education, and were employed) or 0 (if their family had > 4 members, or had no academic education, and were unemployed) was assigned to each of the three variables. These scores were then summed to calculate the participant’s SES score. A score of 3 was considered high SES, a score of 2 was moderate, and a score of 1 or 0 was classified as low SES.

### Statistical analysis

Statistical analysis was conducted using The Statistical Package for Social Sciences (Version 20.0; SPSS, Chicago, IL) (SPSS) software. The normality of the variables in both the case and control groups was assessed using a histogram chart and the Kolmogorov-Smirnoff test. All quantitative variables had normal distribution in the case and control groups because the results of the Kolmogorov-Smirnoff test were not statistically significant (*P* > 0.05), and also the histogram chart visually showed the normality of the distribution of the variables. We compared the mean values of continuous variables using the independent sample t-test. The chi-square test was also used to compare categorical variables. Logistic regression analysis was used to calculate the odds ratios (ORs) and 95% confidence intervals (CIs) for BC across tertiles of different high-protein diets. To account for potential confounding factors, we adjusted the logistic regression model for age, age at first pregnancy, menopausal status, family history of cancer, anti-inflammatory drug use, vitamin D supplementation, physical activity, BMI, SES, and energy intake. We also investigated the association between each of the major subgroups of CHOs, proteins, and fats, including refined and unrefined CHOs, animal and plant proteins, and animal and plant fats, with the risk of BC using logistic regression analysis with adjustment for potential confounding factors. Consequently, the data were analyzed by examining the odds of BC in individuals in the highest tertile compared to the lowest tertile and per increment of one SD of the aforementioned scores. Moreover, we examined how substituting protein with other macronutrients affected the BC risk after adjusting for the potential confounding variables mentioned above. We substituted 50 and 100 kcal of CHO, fat, animal protein, refined CHOs, and animal fat by total and plant protein intakes in the same multivariable logistic regression model. The difference in their coefficients plus their covariance was used to estimate the OR and 95% CI differences. All *P*-values are two-sided and *P*-values < 0.05 were considered statistically significant.

## Results

Participants’ mean ± SD of age and BMI were 47.9 ± 10.3 years and 29.4 ± 5.5 kg/m^2^, respectively. The scores of high-protein-low-CHO and fat diet, high-protein and CHO-low-fat diet, and high-protein and fat-low-CHO diet in participants were 16.5 ± 3.8, 16.5 ± 6.7, and 16.4 ± 5.9, respectively.

Table 1 indicates the baseline characteristics of subjects, including demographic and lifestyle variables, medical history, and dietary intakes in the case and control groups. The participants’ age, first pregnancy age, % of postmenopausal women, and cancer family history were higher in the case group compared to the control group, whereas the anti-inflammatory drug consumption, energy, plant protein, and vitamin D supplement intake in the control group were higher than case group (*P* < 0.05). Also, the high-protein-low-CHO and fat diet score in the control group was higher than the case group (*P* < 0.05). There were no significant differences between cases and controls in other variables.

**Table 1:** Study population characteristics among the breast cancer patients and healthy participants

Variables	Control (*n* = 267)	Case (*n* = 134)	*P*-value
**Demographic data**			
Age (year)	47.1 ± 10.0	49.5 ± 10.7	0.035
First pregnancy age (year)	18.2 ± 7.4	19.6 ± 8.6	0.040
Body mass index (Kg/m^2^)	29.0 ± 5.4	30.1 ± 5.7	0.071
Physical activity (MET/min/week)	32.7 ± 5.2	32.9 ± 5.4	0.701
Menopausal status (yes, %)	42.7	53.7	0.037
Cancer family history (yes, %)	20.6	30.6	0.028
Anti-inflammatory drug (yes, %)	17.2	7.5	0.007
Vitamin D supplement intake (yes, %)	24.3	14.9	0.029
Education level (Bachelor and higher, %)	14.6	19.4	0.178
Occupation (employed, %)	20.6	17.2	0.442
Family size (> 4 members, %,)	55.4	56.7	0.807
Socio economic status (%)			0.531
Low (%)	37.1	38.1	
Middle (%)	43.8	37.3	
High (%)	18.7	21.6	
**Dietary intakes**			
Energy intake (Kcal/d)	2753 ± 798	2562 ± 612	0.015
Carbohydrate (% of energy)	53.0 ± 6.4	52.4 ± 6.1	0.437
Refined CHO (% of energy)	23.8 ± 8.0	24.3 ± 7.8	0.584
Non-refined CHO (% of energy)	30.9 ± 8.0	29.8 ± 8.1	0.215
Protein (% of energy)	12.7 ± 2.1	12.4 ± 2.0	0.100
Animal protein (% of energy)	7.0 ± 2.4	6.8 ± 2.2	0.357
Plant protein (% of energy)	5.9 ± 1.1	5.6 ± 1.1	0.023
Fat (% of energy)	34.3 ± 6.7	35.2 ± 6.6	0.213
Animal fat (% of energy)	11.4 ± 4.4	11.2 ± 3.5	0.684
Plant fat (% of energy)	19.2 ± 7.9	20.3 ± 7.5	0.171
High protein low CHO- fat score	16.8 ± 3.8	15.9 ± 3.9	0.026
High protein-CHO, and low-fat score	16.9 ± 6.8	15.7 ± 6.6	0.087
High protein-fat, and low CHO score	16.5 ± 6.0	16.5 ± 5.7	0.959

Data are expressed as mean ± SD and percent (%) for continuous and categorical variables, respectively

*CHO* carbohydrate, *MET* metabolic equivalent

Table 2 reported the ORs (95%CI) of BC based on the different high protein scores (including high-protein-low CHO and fat diet score, high-protein and CHO-low-fat diet, and high-protein and fat-low-CHO diet) in tertiles and a per increment of one SD among the study population. In the fully adjusted model after adjusting for age, first pregnancy age, menopausal status, family history of cancer, anti-inflammatory drug use, Vitamin D supplementation, physical activity, body mass index, socio-economic status, and energy intake, individuals in the third tertile of high-protein-low-CHO and fat diet score had lower odds of BC (OR: 0.48, 95%CI: 0.27–0.85, P-trend: 0.008) compared with those in the first tertile. However, based on the multivariable model, no significant association was found between the scores of high-protein and CHO-low-fat diet (OR: 0.58, 95%CI: 0.33–1.01, P-trend: 0.061) and high protein and fat-low-CHO diet with odds of BC (OR: 0.91, 95%CI: 0.52–1.59, P-trend: 0.665). Also, Table 2 showed that the odds of BC decreased by 29% (OR: 0.71, 95%CI: 0.56–0.90) with each SD increase in the high-protein-low-CHO and fat diet score. Furthermore, each SD increment in the high-protein and CHO-low-fat score was associated with 24% decreased odds of BC (OR: 0.76, 95%CI: 0.60–0.97) in the final model.

**Table 2:** The odds ratio (95% confidence interval) of breast cancer based on the different high protein scores in tertiles or per increment of one standard deviation among the study population

	OR of breast cancer (95% CI)					
	T1	T2	T3	P trend	Per one SD	*P*-value
**High-protein-low-CHO and fat diet**						
Median score, SD	12.0	17.0	21.0	-	3.83	-
Case/total	58 / 138	36 / 133	40 / 130	-	134 / 401	-
Model 1^a^	1.00 (Ref)	0.52 (0.31–0.89)	0.65 (0.39–1.08)	0.068	0.80 (0.65–1.00)	0.045
Model 2^b^	1.00 (Ref)	0.45 (0.26–0.78)	0.58 (0.34–1.00)	0.031	0.77 (0.61–0.96)	0.022
Model 3^c^	1.00 (Ref)	0.39 (0.22–0.69)	0.48 (0.27–0.85)	0.008	0.71 (0.56–0.90)	0.005
**High-protein and CHO-low-fat score**						
Median score, SD	10.0	18.0	24.0	-	6.73	-
Case/total	58 / 156	42 / 118	34 / 127	-	134 / 401	-
Model 1^a^	1.00 (Ref)	0.96 (0.57–1.60)	0.66 (0.39–1.11)	0.143	0.85 (0.69–1.06)	0.146
Model 2^b^	1.00 (Ref)	0.91 (0.53–1.55)	0.63 (0.36–1.10)	0.115	0.81 (0.64–1.02)	0.070
Model 3^c^	1.00 (Ref)	0.84 (0.49–1.45)	0.58 (0.33–1.01)	0.061	0.76 (0.60–0.97)	0.026
**High-protein and fat-low-CHO score**						
Median score, SD	10.0	17.0	23.0	-	5.92	-
Case/total	49 / 139	43 / 135	42 / 127	-	134 / 401	-
Model 1^a^	1.00 (Ref)	0.82 (0.49–1.37)	0.89 (0.53–1.50)	0.631	0.99 (0.80–1.22)	0.913
Model 2^b^	1.00 (Ref)	0.72 (0.42–1.24)	0.91 (0.52–1.58)	0.655	0.99 (0.79–1.24)	0.941
Model 3^c^	1.00 (Ref)	0.73 (0.42–1.25)	0.91 (0.52–1.59)	0.665	0.99 (0.79–1.24)	0.908

*CHO* Carbohydrate

^a^Model 1: adjusted for age, first pregnancy age

^b^Model 2: adjusted for model 1 and menopausal status, family history of cancer, anti-inflammatory drug use, Vitamin D supplementation, physical activity, body mass index, and socio-economic status

^c^Model 3: adjusted for model 2 and energy intake

Table 3 expresses the substitute analysis for the association of replacing macronutrients together on the odds of BC, calculated using logistic regression models. Each 50 or 100 kcal replacement of fat by protein was associated with 20% (95%CI_difference_: -0.36, -0.03) and 40% (95%CI_difference_: -0.73, -0.07) lower odds of BC in participants, respectively. Also, the odds of BC were decreased by 33% (95%CI_difference_: -0.63, -0.03) with each 50 kcal and by 66% (95%CI_difference_: -1.26, -0.07) with each 100 kcal replacement of plant protein instead of refined CHO. Regarding the replacement of other macronutrients with each other, no significant was observed.

**Table 3:** Substitution analysis for the association of replacing macronutrients together on the odds of breast cancer calculated using logistic regression models^a^

Substituting X instead of Y	per 50 Kcal		per 100 Kcal	
	OR differences (95% CI)	*P*-value	OR differences (95% CI)	*P*-value
**Protein** instead of **CHO**	-0.10 (-0.31, 0.10)	0.325	-0.21 (-0.63, 0.21)	0.325
**Protein** instead of **fat**	-0.20 (-0.36, -0.03)	0.016	-0.40 (-0.73, -0.07)	0.016
**Plant protein** instead of **animal protein**	-0.14 ( -0.44. 0.15)	0.351	-0.28 (-0.88, 0.31)	0.351
**Plant protein** instead of **refined CHO**	-0.33 (-0.63, -0.03)	0.028	-0.66 (-1.26, -0.07)	0.028
**Plant protein** instead of **animal fat**	-0.20 (-0.46, 0.05)	0.126	-0.40 (-0.92, 1.11)	0.126

*CHO* Carbohydrate

^a^All analyses were adjusted for age, first pregnancy age, menopausal status, family history of cancer, anti-inflammatory drug use, vitamin D supplementation, physical activity, body mass index, socio-economic status, and energy intake

**Fig. 1:**
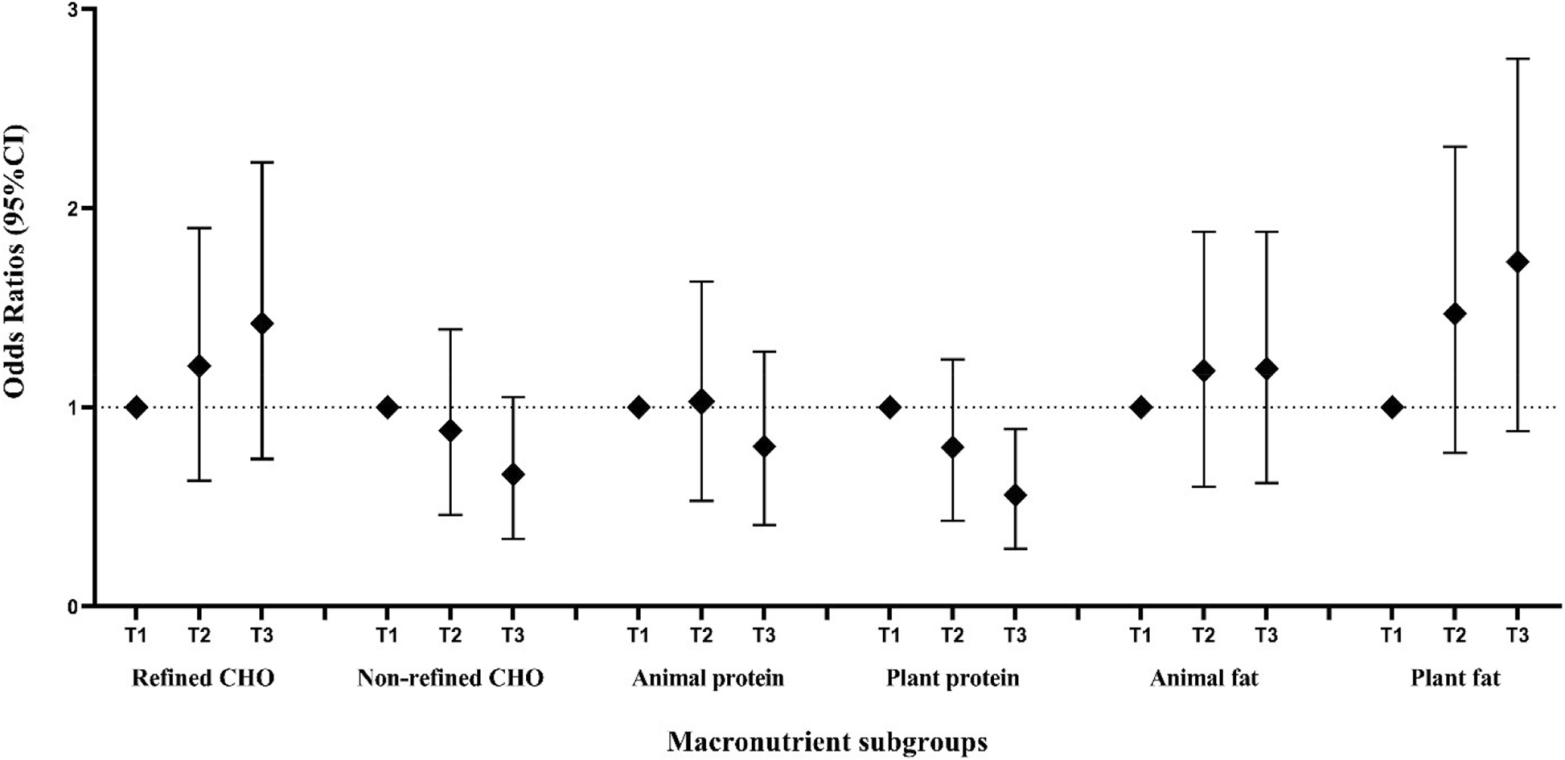
The odds ratio (95% confidence interval) of breast cancer according to tertiles of 6 subgroups of macronutrient intake using the multivariable model adjusted for age, first pregnancy age, menopausal status, family history of cancer, anti-inflammatory drug use, vitamin D supplementation, physical activity, body mass index, socio-economic status, and energy intake

Figure 1 shows the association of 6 subgroups of macronutrient intake with the odds of BC in our study population. After adjusting for potential confounding variables, including age, first pregnancy age, menopausal status, family history of cancer, anti-inflammatory drug use, vitamin D supplementation, physical activity, body mass index, socio-economic status, and energy intake, participants in the highest tertile of plant protein intake had 50% (OR: 0.50, 95%CI: 0.29–0.89, P-trend: 0.018) decreased odds of BC than those in the lowest tertile. However, no significant association was observed between refined-CHO, non-refined-CHO, plant fats, animal fats, and animal proteins and the risk of BC.

## Discussion

The present study showed that higher adherence to diets with high-protein-low-CHO and fat score and high-protein and CHO-low-fat score were inversely associated with odds of BC. However, high-protein and fat-low CHO diet was not related to BC risk. In addition, our substitution analysis reported that substituting fats with protein or replacing refined CHO with plant protein in the diet of participants may contribute to decreasing the risk of BC.

To the best of our knowledge, our investigation is the first to examine the association between different high protein diet scores and the risk of BC in the framework of a case-control study among Iranian adults. In recent decades, many studies investigated the relationship between dietary intake of macronutrients and the risk of BC. In accordance with our findings, several randomized clinical trials (RCTs) reported that a low-fat dietary pattern significantly reduced the incidence of ovarian cancer [36] and the risk of BC mortality [37], however, other RCTs observed no significant association between a low-fat dietary pattern with risk of breast [38] and colorectal cancer [39]. In line with our study results, an observational study conducted in the US population shows that higher plant protein intake especially protein from vegetables was associated with lower BC incidence [24]. Also, the findings of the present, are in agreement with the results of a large prospective study that has reported higher intake of plant protein was associated with decreased cardiovascular and total mortality, and plant protein intake instead of red meat protein or processed meat protein was related to lower cancer-related, cardiovascular-related and total mortality [40]. Also, aligned with our findings, a meta-analysis of prospective studies reported higher total protein intake was related to a decreased risk of all-cause mortality, and plant protein intake was associated with decreased risk of all-cause and cardiovascular disease mortality. Also, this study shows that consumption of plant protein sources instead of animal protein could be associated with longevity [41]. Furthermore, an animal study shows that a diet with low-CHO, and high-protein decreases tumor growth and prevents cancer initiation [42]. However, contrary to our results, in the Nilsson. et al. study no significant association was observed between the higher low-CHO, and high-protein scores and the risk of BC [43]. Also, Chow et al. reported high protein intake has been related to the development of other chronic renal conditions that may increase renal cell cancer. Furthermore, two cohort studies indicated a low CHO-high protein diets were associated with increased risk of cardiovascular diseases [44] and total mortality [45] in Swedish women.

In the current study, we showed that replacing refined CHO with plant protein and replacing fats with protein in the diet can reduce the risk of BC. In other words, our findings support the idea that if a high-protein diet is based on plant-based food choices, it can be useful in preventing the occurrence of chronic diseases, such as BC; This is because a high-protein- low-fat diet based on plant-based food intakes emphasizes high consumption of legumes, whole grains, seeds, nuts, and soy products, and lower consumption of red and processed meat, refined grains, sweetened beverages, and high-fat foods. Adhering to this dietary pattern means high intakes of plant proteins, micronutrients with antioxidant properties, and fiber and less intakes of saturated fat, simple sugar, and salt. Such a dietary pattern can provide antioxidant [46], anti-inflammatory [47], and anti-insulin resistance properties [48] that can help protect individuals against the risk of BC [42]; therefore, it is expected that if individuals follow a high-protein-low-fat and CHO diet based on plant food choices, they can better protect themselves against the risk of BC. It should be noted that plant food sources containing high protein, such as whole grains, legumes, and seeds, are considered rich sources of complex CHOs. Therefore, in this case, a plant-based high-protein diet can be regarded as a high-CHO diet, all while still being known as a low-fat diet. The relationship between this type of diet and the risk of chronic diseases, such as BC, may also be inverse; our study confirms this claim, as we discovered that following a high-protein and CHO-low fat diet can be linked to a reduced risk of BC.

The mechanisms underlying the role of diet with different high protein scores and BC risk are not yet fully understood. It seems a diet with a high protein and low-fat score especially protein derived from plant sources, regardless of CHO intake, leads to a greater intake of fiber, vitamins, minerals, and polyphenols which can be effective in decreasing the risk of BC [10, 49–51]. Simple CHOs as a possible factor in increasing the BC risk, make up the majority of CHO intake among the Iranian population and replace it with plant proteins such as legumes that have possible anti-carcinogenic effects [52]. The reason for this result may be that plant foods such as soy contain fibers and phytoestrogens that induce apoptosis [53]. Animal protein intake is usually associated with the intake of fat which may throughout carcinogenic heterocyclic amines lead to an increased risk of cancer [54]. Also, Taha et al. hypothesized that a diet with high casein might increase the progression of cancer cells in mice through the activation of the IGF/Akt/mTOR pathway [55]. Also, it seems proteins from animal sources throughout increased insulin-like growth factor-1 [56], and the expression of Ras homologous gene family member A and vascular endothelial growth factor receptor-2 [57] lead to tumor progression. However, plant protein intake was inversely associated with RhoA expression [57].

Our study has several strengths. To our knowledge, this is the first study on the association between different high-protein diet scores (high-protein-low-CHO and fat diet, high-protein and CHO-low-fat diet, and high-protein and fat-low-CHO diet) and the risk of BC in the Iranian population. Also, this study included a substitution analysis in which we examined the effects of substituting protein with other macronutrients on BC risk while controlling for potential confounding variables. This represents the first time this type of analysis has been conducted in relation to protein intake and BC. In addition, we used validated questionnaires to collect individual data on dietary intake and physical activity levels, which minimized the possibility of recall bias. We included the patients that newly diagnosed with BC (< 6 months) in the case group; therefore, the individuals included in the current study possibly had not changed their usual lifestyle (including diet and physical activity) due to their chronic illness. Finally, we tried to control the effect of various potential confounding variables in assessing the relationship between different high-protein diet scores and the risk of BC, as much as possible. Some limitations of the present study should be reported. First, recall bias and selection bias are difficult to avoid in case-control studies. Second, regarding the nature of case-control design, investigating the causality relationship in this study is impossible. Third, since alcoholic drinks such as wine and beer are not common or may be unreported in the Iranian population due to religious considerations and legal restrictions; therefore we could not determine participants’ data for alcohol consumption, which could have played a confounding role in the present study. Fourth, the effect of some variables that were unknown to us may not have been controlled in our statistical analyses. Finally, using questionnaires to collect dietary intake and physical activity information may cause measurement errors and recall bias, but to decline the errors we used validated and reliable questionnaires which were specially developed for the Iranian population.

## Conclusion

In conclusion, our findings revealed that a high-protein-low-CHO and fat diet based on plant-based food choices can be associated with a reduced risk of BC. Also, the current study suggested that higher plant protein intake especially instead of fats and refined CHO in individuals’ diet can be considered for prevention of BC risk. Further dietary intervention trials, prospective and longitudinal studies are recommended to address the role of the different high protein diet scores in the prediction of BC risk and the mechanisms justifying this possible relationship. This finding is very important since it can help public health and be considered as a recommendation in dietary guidelines which a diet with high-protein-low-CHO and fat diet based on plant-based food choices, can easily prevent the occurrence of BC.

## Data Availability

The datasets analyzed in the current study are available from the corresponding author on reasonable request.

## Abbreviations

BC: Breast cancer
BMI: Body mass index
CHO: Carbohydrate
CI: Confidence interval
FCT: Food composition table
FFQ: Food frequency questionnaire
HRT: Hormone replacement therapy
MET: Metabolic Equivalents
OCP: Oral contraceptive pill
OR: Odds ratio
RCT: Randomized clinical trial
SD: Standard deviation
SES: Socio-economic status
SPSS: Statistical Package for Social Sciences
USDA: United States Department of Agriculture
